# Deep learning generates synthetic cancer histology for explainability and education

**DOI:** 10.1038/s41698-023-00399-4

**Published:** 2023-05-29

**Authors:** James M. Dolezal, Rachelle Wolk, Hanna M. Hieromnimon, Frederick M. Howard, Andrew Srisuwananukorn, Dmitry Karpeyev, Siddhi Ramesh, Sara Kochanny, Jung Woo Kwon, Meghana Agni, Richard C. Simon, Chandni Desai, Raghad Kherallah, Tung D. Nguyen, Jefree J. Schulte, Kimberly Cole, Galina Khramtsova, Marina Chiara Garassino, Aliya N. Husain, Huihua Li, Robert Grossman, Nicole A. Cipriani, Alexander T. Pearson

**Affiliations:** 1 Section of Hematology/Oncology, Department of Medicine, University of Chicago Medicine, Chicago, IL USA; 2 Department of Pathology, University of Chicago Medicine, Chicago, IL USA; 3 Tisch Cancer Institute, Icahn School of Medicine at Mount Sinai, New York, NY USA; 4 Ghost Autonomy, Inc., Mountain View, CA USA; 5 Department of Pathology and Laboratory Medicine, University of Wisconsin at Madison, Madison, WN USA; 6 University of Chicago, Center for Translational Data Science, Chicago, IL USA

**Keywords:** Cancer, Mathematics and computing

## Abstract

Artificial intelligence methods including deep neural networks (DNN) can provide rapid molecular classification of tumors from routine histology with accuracy that matches or exceeds human pathologists. Discerning how neural networks make their predictions remains a significant challenge, but explainability tools help provide insights into what models have learned when corresponding histologic features are poorly defined. Here, we present a method for improving explainability of DNN models using synthetic histology generated by a conditional generative adversarial network (cGAN). We show that cGANs generate high-quality synthetic histology images that can be leveraged for explaining DNN models trained to classify molecularly-subtyped tumors, exposing histologic features associated with molecular state. Fine-tuning synthetic histology through class and layer blending illustrates nuanced morphologic differences between tumor subtypes. Finally, we demonstrate the use of synthetic histology for augmenting pathologist-in-training education, showing that these intuitive visualizations can reinforce and improve understanding of histologic manifestations of tumor biology.

## Introduction

Accurate diagnosis from histopathology is the first step in the evaluation of many cancers, with management pivoting upon a tumor’s morphologic classification. In addition to morphologic assessment, molecular profiling through analysis of DNA mutations, RNA fusions, and gene expression is also increasingly utilized, as a tumor’s molecular subtype may inform prognosis or allow targeted therapies. Deep neural networks (DNN), a form of artificial intelligence, can classify tumors from pathologic images with high accuracy, and several studies have shown that these models can also detect actionable genetic alterations and gene expression from tumor histology even when the associated histopathological phenotype is unknown^1^. Although still in their nascent stages, DNN applications in digital pathology are being explored to assist with technical tasks, automate or augment pathologist workflows, and extend pathologist capabilities through the development of novel biomarkers^2^. DNNs are however limited by their lack of predictive transparency, which is contributing to an explainability crisis as the scientific community attempts to interpret these frequently opaque models^3,4^. When a neural network trained to detect molecular subtype performs well but the corresponding histologic features are poorly understood, explainability tools may provide insights into what the model learned and help ensure predictions are based on biologically plausible image features.

Many techniques exist for explaining artificial intelligence models in medical image analysis, as recently outlined by van der Velden et al. ^5^. The most common explainability approaches use visual explanations that highlight areas of an image important to the final prediction. These local explainability methods, which include saliency mapping^6^, attention^7^, and perturbation-based approaches^8^, provide insights into how a prediction was made for a specific image, contrasting with global explainability methods that provide dataset-level insights into image features learned during training. Their visual explanations are attractive due to ease of interpretability, although concerns have been raised that localizations from these techniques may not be entirely accurate for medical imaging applications^9,10^. Localization-based approaches are most helpful in instances where predictions can be attributed to a discrete object, but medical images – particularly histopathological images, which are largely textural – may not manifest predictive features amenable to clear localization. The reliability of these methods in identifying textural image features with predictive significance is unclear.

Many other approaches to providing explanations for deep learning model predictions are similarly limited for histopathological applications. Image captioning methods seek to provide text-based explanations through clearly interpretable, plain language^11^, but these approaches require additional ground-truth text labels for training, and it is not clear how such approaches would translate to DNN histopathological models. Testing with concept activation vectors (TCAV) provides explanations through identifying which concepts in an image are most relevant for the prediction^12^, but this approach similarly requires an additional labeled dataset and is limited to providing explanations from only prespecified concepts. Example-based explanations provide a collection of sample images similar to the specified image through analysis of neighbors in the classifier latent space^13^, but do not offer insights into specific image features important to the prediction.

Generative adversarial networks (GAN) are deep learning models that use a pair of competing neural networks, called the generator and discriminator, to create realistic images. Conditional GANs (cGANs) use additional information to control the generation process, providing the ability to create images belonging to a particular class or style and smoothly transition between classes^14,15^. Recent work has shown that cGANs can be leveraged as a tool for explaining DNN classifiers, providing image-specific explanations that offer dataset-level insights into differences between image classes^16,17^. As an explainability tool, cGANs yield easily interpretable, visually clear explanations that differ from other approaches in that they are not limited to explaining localizable image features and do not require additional labeled training data. This dataset-level explainability method offers a fundamentally different, yet complementary, approach to explainability than is typically used in medical imaging explications. Rather than answering the question “why has a certain prediction been made for a specific image?”, this method helps answer the question “what image features are associated with each class?”.

A growing body of evidence is showing that GANs can create realistic histologic images^18–24^. Quiros et al. showed that GANs can generate realistic artificial cancer tissue, and that traversal of the GAN latent space results in realistic images with smooth architectural changes. Levine et al. used a cGAN to generate synthetic histologic images indistinguishable from real images, which were accurately classified by both human pathologists and DNNs trained on real images. Krause et al. demonstrated that synthetic images generated by a cGAN improve detection of genetic alterations in CRC when used for training augmentation^21^, and other groups have similarly shown that augmentation with images generated from cGANs can improve classifier performance^22,25,26^. Finally, several groups have explored the use of GANs for stain and color normalization^27–29^, virtual staining^30–32^, and image enhancement^33–36^. Their potential utilization as an explainability tool for histopathological DNN models, however, remains unexplored.

Here, we describe an approach to explaining histopathological DNN models using cGAN-generated synthetic histology. We show that cGAN-generated synthetic histology provides visually clear, dataset-level insights into the image features associated with DNN classifier predictions. Furthermore, we demonstrate that generation of synthetic histology can be fine-tuned through class and layer blending to provide nuanced insights into the histologic correlates for a given tumor subtype or molecular state at varying scales. Finally, we show that synthetic histologic visualizations are sufficiently intuitive and informative to improve pathology trainee classification of a rare tumor subtype.

## Results

**Fig. 1:**
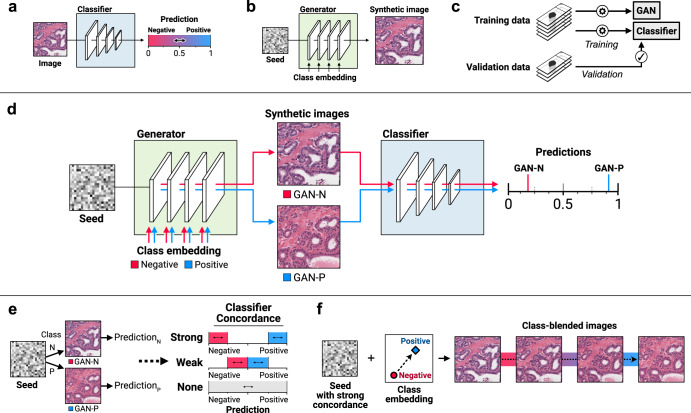
cGAN-based approach for explaining histopathological models with synthetic histology. **a** Our approach starts with a classification model trained to predict a molecular outcome from a histologic image. **b** A separate conditional generative adversarial network (cGAN) model is then trained to generate synthetic histologic images. cGANs create synthetic images from a seed of random noise and a class label, passed to each layer in the network through an embedding. **c** The same training data is used for training both the classifier and cGAN. Held-out validation data is used to validate the predictive accuracy of the classifier. **d** The trained cGAN and classifier are then combined to form a single pipeline. For a given seed, synthetic images are generated for both the negative and positive classes. Both images are then stain normalized and passed to the classifier, resulting in two predictions. If the classifier predictions match the synthetic image labels, the seed is designated “classifier-concordant” and the synthetic images can be used for morphologic explanations. Visualizing classifier-concordant image pairs side-by-side allows one to appreciate the histologic features associated for classifier predictions, providing a tool for model explainability and education. **e** Classifier-concordant seeds are further subdivided into weakly- and strongly-concordant based on the magnitude of the predictions. **f** Class-blended images are generated for strongly-concordant seeds by interpolating between class embeddings while holding the seed constant.

Our approach starts with training DNN classification and cGAN models on digital pathology images (Fig. 1a–c). cGANs generate an image from the combination of a seed – a vector of random numbers that determines what the image will broadly look like – and a class label, which influences the image toward one class or another. The class label is converted into an embedding, a vector of numbers learned through training that encodes the essence of the class, and passed to each layer of the cGAN. For a given seed, synthetic histologic images and corresponding classifier predictions are generated for each class (Fig. 1d). If the predictions are strong and match the cGAN class label, the seed has strong classifier concordance, and if the predictions are weak but match the cGAN class label, the seed has weak concordance (Fig. 1e). Side-by-side, classifier-concordant image pairs illustrate histologic differences responsible for changes in classifier prediction, assisting with model explainability. To create an image in transition from one class to another, we perform a linear interpolation between two class embeddings and use the interpolated embedding for cGAN conditioning; using the same seed but gradually interpolating the embedding creates class-blended images that gradually shift from one class to another (Fig. 1f).

### Non-small cell lung cancer subtyping

**Fig. 2:**
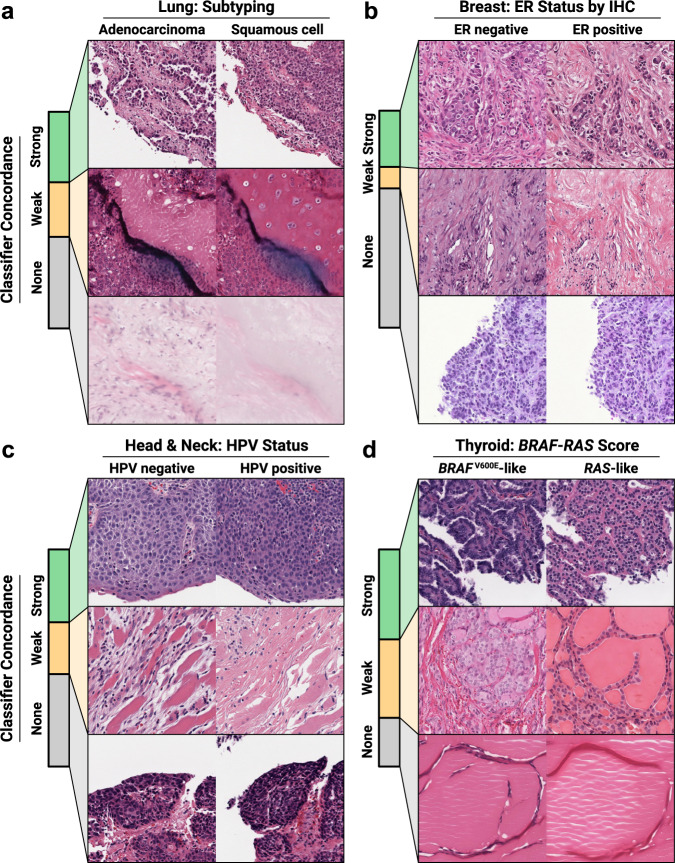
Synthetic histology illustrates molecular states expressed in tumor histopathology. **a** A cGAN was trained on lung adenocarcinoma vs. squamous cell carcinoma. Classifier concordance for 1000 seeds was 31.1% strong, 27.0% weak, and 41.9% non-concordant. **b** A second cGAN was trained on breast cancer estrogen receptor (ER) status determined by immunohistochemistry (IHC). Classifier concordance for 1000 seeds was 25.9% strong, 10.0% weak, and 64.1% non-concordant. **c** A third cGAN was trained on head and neck cancer Human Papillomavirus (HPV) status, as determined by either PCR or p16 immunohistochemical staining. Classifier concordance for 1000 seeds was 33.6% strong, 23.9% weak, and 42.6% non-concordant. **d** A final cGAN was trained on thyroid neoplasm *BRAF-RAS* gene expression score (BRS). Classifier concordance for 1000 seeds was 41.2% strong, 36.2% weak, and 22.6% non-concordant.

We trained a classifier and cGAN for non-small cell lung cancer (NSCLC) conditioned on adenocarcinoma vs. squamous cell carcinoma, as this is a well-described histologic phenotype suitable for assessing feasibility of the approach when the expected morphologic differences are known (Fig. 2a and Supplementary Fig. 1). The classifier and cGAN models were trained on the same dataset of 941 slides from The Cancer Genome Atlas (TCGA). Three-fold cross-validation Area Under Receiver Operator Curve (AUROC) for the classifier was 0.96 ± 0.01, with an AUROC of 0.98 (95% CI 0.97 – 1.0) on an external test set of 1306 slides from the Clinical Proteomics and Tumor Analysis Consortium (CPTAC) (Supplementary Table 1). The trained cGAN was evaluated by calculating Fréchet Inception Distance (FID)^37^, a commonly used metric to evaluate realism and diversity of GAN-generated images due to its sensitivity to distributional changes and consistency with human evaluation. Lower FID values indicate higher quality images, and for comparison, FID values for highly-performing GANs reported in the StyleGAN papers^14,15,38^ range from 3-6. FID for the lung cGAN was 3.67. Expert pathologist assessment revealed that strongly-concordant synthetic images were realistic and consistent with the cGAN class labels (Supplementary Fig. 2). The synthetic image pairs illustrated known histologic differences in adenocarcinomas and squamous cell carcinomas, including gland formation, micropapillary morphology, and papillary projections in the adenocarcinoma images, and intercellular bridging and keratinization in the squamous cell images. Some strongly concordant seeds, however, did not clearly illustrate diagnostic-grade differences between image pairs. For example, some image pairs lacked tumor, instead illustrating differences in level of necrosis, which was increased in squamous cell images, or non-diagnostic stromal changes, with an orange tint seen in some squamous cell images.

### Estrogen receptor status in breast cancer

We repeated the approach for the molecular outcome of breast cancer estrogen receptor (ER) status, chosen because ER status influences morphologic phenotype on standard hematoxylin and eosin (H&E) stained slides^39,40^, but the morphologic correlates are incompletely characterized (Fig. 2b). Classification and cGAN models were trained on 1048 slides from TCGA, and the classifier was externally validated on a dataset of 98 slides from CPTAC. Three-fold cross-validation AUROC was 0.87 ± 0.02 for the classifier, with a test set AUROC of 0.81 (95% CI 0.72–0.90) (Supplementary Table 1). FID for the trained cGAN was 4.46. Two expert breast pathologists concluded that the synthetic ER-negative images had higher grade, more tumor-infiltrating lymphocytes, necrosis, and/or apocrine differentiation compared with ER-positive images, consistent with known histopathological associations with ER status (Supplementary Fig. 3).

### Human papillomavirus status in head and neck cancer

We next tested the approach for the molecular outcome of Human Papillomavirus (HPV) status in head and neck cancer (Fig. 2c). HPV status is known to influence morphologic phenotype on standard H&E slides, but PCR or IHC is required for diagnosis. We trained classification and cGAN models on a single-site institutional dataset of 362 slides, with HPV status determined through positivity by either PCR or p16 IHC. The classification model was externally validated on a dataset of 405 slides from TCGA. Three-fold cross-validation AUROC was 0.83 ± 0.05 for the classifier, with a test set AUROC of 0.83 (95% CI 0.77 – 0.89) (Supplementary Table 1). FID for the trained cGAN was 7.35. Two pathologists reviewed the synthetic histology explanations, concluding that HPV-negative images showed greater keratinization, intercellular desmosomes, increased cytoplasm, and pleomorphic nuclei, compared with HPV-positive images that showed increased inflammation, tightly packed syncytial cells with decreased cytoplasm, and smaller, more monotonous nuclei, consistent with known histopathologic associations^41,42^ (Supplementary Fig. 4).

### BRAF-RAS gene expression in thyroid neoplasms

**Fig. 3:**
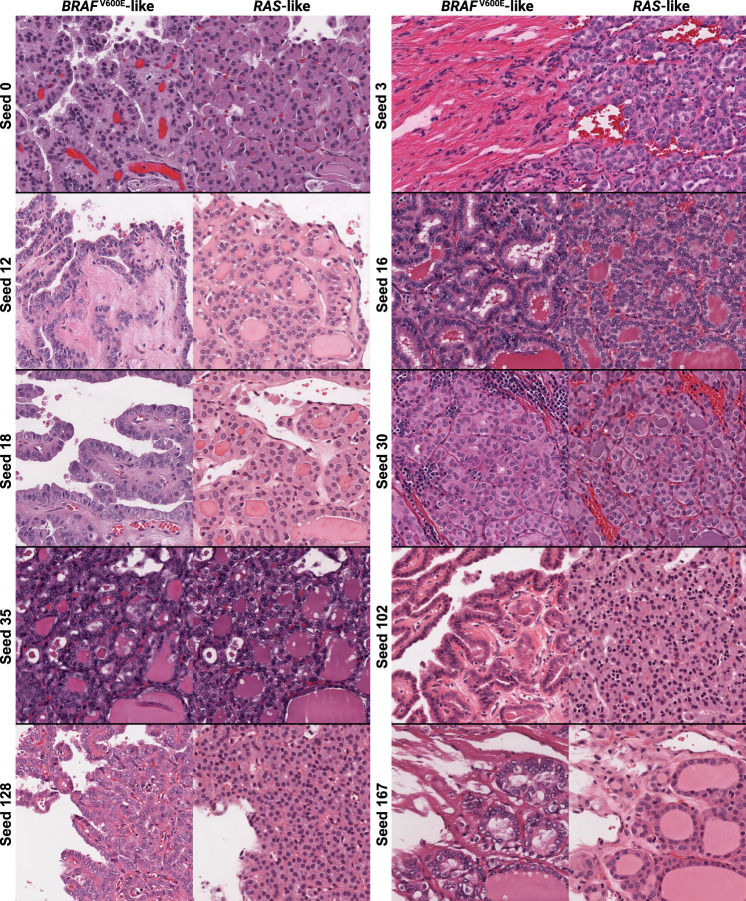
cGAN-generated synthetic histology illustrates morphologic differences associated with *BRAF*-*RAS* gene expression score in thyroid neoplasms. Classifier-concordant seeds from the thyroid cGAN were reviewed with an expert thyroid pathologist and pathology fellow to determine thematic differences in cGAN-generated *BRAF*^V600E^-like and *RAS*-like histologic features. Seed 0 illustrates architectural differences, with a papillae in the *BRAF*^V600E^-like image replaced with colloid in the *RAS*-like image. Seed 3 highlights the an increase in fibrosis in the *BRAF*^V600E^-like image. The *BRAF*^V600E^-like image for seed 12 shows a cystic structure with cell lining, which is replaced with what appears to be a tear in the *RAS*-like image, accompanied by architectural differences moving from papillae in the *BRAF*^V600E^-like image to follicles in the *RAS-*like image. Seed 16 demonstrates an increase in lacunae caused by resorbed colloid in the *BRAF*^V600E^-like image, compared with smaller, more regular follicles in the *RAS*-like image. Seed 18 shows papillae, a papillary vessel, and a cystic structure in the *BRAF*^V600E^-like image replaced with follicles, colloid, and an endothelial-lined vessel in the *RAS*-like image, respectively. Seed 30 highlights more tumor-infiltrating lymphocytes in the *BRAF*^V600E^-like along with increased cytoplasmic density compared with the *RAS*-like image. Seed 35 shows overall similar architecture in the two images, but with greater cell flattening in the *RAS*-like image compared to the *BRAF*^V600E^-like image. Seeds 102 and 128 both illustrate nuclear pleomorphism, increased cytoplasm, papillae, and scalloping in the *BRAF*^V600E^-like images compared with the *RAS*-like image. Seed 167 highlights nuclear pleomorphism and fibrosis in the *BRAF*^V600E^-like image compared with *RAS*-like image which has monotonous, circular, non-overlapping nuclei with regular contours and fine, dark chromatin.

**Fig. 4:**
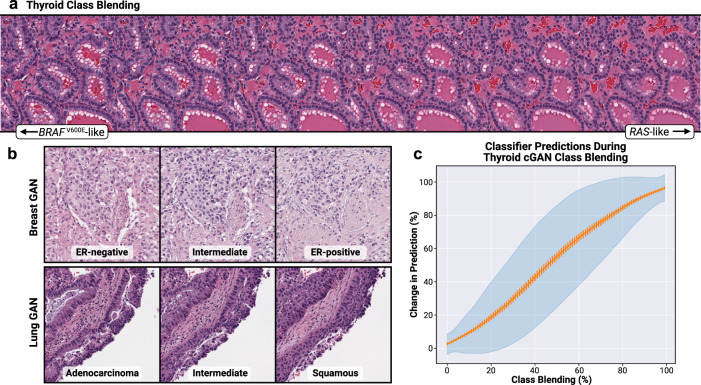
Class blending yields smooth histologic transitions between classes. **a** Illustration of class blending performed for a strongly-concordant Thyroid cGAN seed, transitioning from *BRAF*^V600E^-like to *RAS*-like. Intermediate images are generated through linear interpolation between class embeddings. **b** Example class-blended images for the Lung and Breast cGANs. **c** Classifier predictions smoothly transition during class blending. For 1000 strongly-concordant seeds, predictions were generated for synthetic images during class blending, transitioning from *BRAF*^V600E^-like to *RAS*-like. For each seed, predictions were assessed at 100 intermediate steps during class blending. The orange line indicates the proportion of change in prediction at each step, from *BRAF*^V600E^-like to *RAS*-like, during class blending for all 1000 seeds. The orange bars indicate the 95% confidence interval of the average change in prediction across all seeds at each class blending step. The blue shaded interval represents the standard deviation.

Finally, we trained a cGAN on thyroid neoplasms, conditioned on the molecular outcome of whether the tumor had *BRAF*^V600E^-like or *RAS*-like gene expression (Fig. 2d). *BRAF-RAS* gene expression score (BRS), a score between -1 (*BRAF*^V600E^-like) and +1 (*RAS*-like), correlates with thyroid neoplasm histologic phenotype and can be used to distinguish malignant papillary thyroid carcinomas (PTC) from the low-risk non-invasive follicular thyroid neoplasms with papillary-like nuclear features (NIFTP), despite the fact that these entities are challenging to distinguish even by experienced pathologists^43–46^. We trained a DNN regression model to predict BRS as a linear outcome and evaluated performance as a classifier by discretizing the predictions at 0. The classification and cGAN models were trained on 369 WSIs from TCGA, and the classifier was externally validated on an institutional dataset of 134 tumors, including 76 *BRAF*^V600E^-like PTCs and 58 *RAS*-like NIFTPs, as previously reported^43^. Three-fold cross-validation AUROC was 0.94 ± 0.03, with an external test set AUROC of 0.97 (95% CI 0.95–1.0) (Supplementary Table 1). The cGAN generated realistic and diverse images with an FID of 5.19. cGAN visualizations illustrate subtle morphologic changes associated with the *BRAF-RAS* spectrum, including nuclear changes (enlargement, chromatin clearing, membrane irregularities), architectural changes (elongated follicles, papillae), colloid changes (darkening, scalloping), and stromal changes (fibrosis, calcification, ossification) (Fig. 3). Class blending provides realistic histologic images that gradually transition from *BRAF*^V600E^-like morphology to *RAS*-like morphology, and predictions of these blended images smoothly change from *BRAF*^V600E^-like to *RAS*-like (Fig. 4).

### Effect of classifier architecture and stain normalization

To investigate the potential effect of classifier architecture selection on the cGAN explainability pipeline, we trained additional classifiers on the thyroid *BRAF-RAS* gene expression endpoint using the MobileNetV2^47^, ResNet18^48^, and EfficientNet-B3^49^ architectures. AUROC on the external test set was similar between architectures, at 0.97 (95% CI 0.95–1.00), 0.96 (95% CI 0.93–0.99), and 0.98 (0.96–1.00), respectively (Supplementary Fig. 5). When used for determining classifier concordance from the trained cGAN, selected strongly-concordant patches were highly similar between Xception and EfficientNet-B3, with 41.2% and 42.6% seeds selected by each classifier, respectively. The classifier concordant seeds identified between these two architectures exhibited high overlap, with 90.9% of classifier concordant seeds selected by both classifiers. MobileNetV2 and ResNet18 both selected fewer strongly concordant image patches, with 31.6% and 27.0% seeds identified as strongly concordant, respectively. However, there was still a strong degree of overlap between classifier concordant seeds; 89.5% of classifier-concordant seeds by MobileNetV2 were also classifier-concordant by Xception, and 90.0% of ResNet18 classifier-concordant seeds overlapped with Xception concordant seeds. Taken together, Xception and EfficientNet-B3 reproduced highly similar seeds when selecting synthetic images for explainability. Smaller architectures, such as MobileNet-V2 and ResNet18, labeled fewer seeds as strongly concordant, but the selected classifier-concordant seeds were highly similar to the seeds also selected by Xception and EfficientNet-B3.

Digital stain normalization methods are commonly used to assist with reducing the effect of varying staining intensities when training deep learning models on digital pathology images. All classifiers were trained with a modified Reinhard algorithm, but cGANs were trained on non-normalized images to avoid unrealistic colors or artifacts caused by normalization that may interfere with pathologist interpretation. Selected synthetic histology images are shown before and after stain normalization in Supplementary Fig. 6. We investigated the effect of different stain normalization strategies on classifier performance and assessment of classifier concordance by comparing four normalization strategies, and all four strategies yielded similar performance on the external test sets (Supplementary Fig. 7 and Supplementary Table 2). For the *BRAF-RAS* gene expression endpoint, 92.1% of classifier-concordant seeds as determined by a classifier trained on the Reinhard method were also selected by the classifier trained using the modified Reinhard method. Similarly, 92.7% of seeds selected by the Macenko classifier and 90.0% of seeds selected by a classifier trained without stain normalization were also selected by the standard classifier trained with the modified Reinhard method. In summary, the choice of stain normalization method had little effect on classifier performance, and all methods yielded similar classifier-concordant seeds for downstream explainability.

### cGAN layer blending

**Fig. 5:**
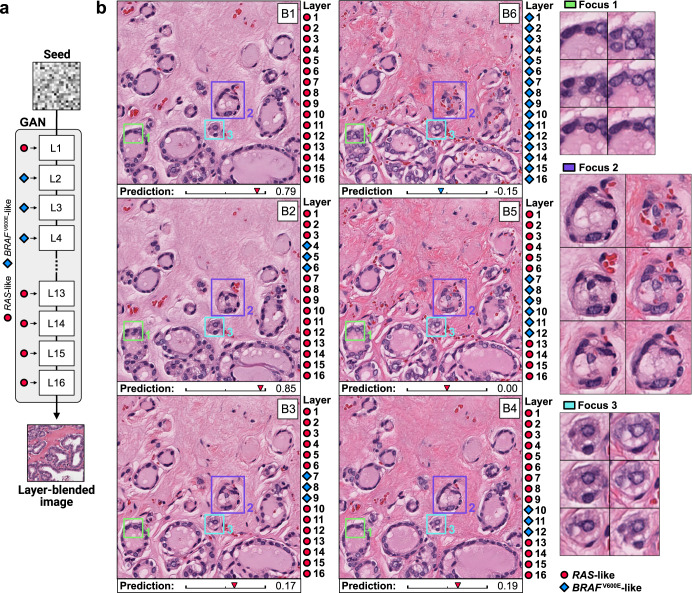
Class and layer blending provides nuanced insights into *BRAF*^V600E^-like and *RAS*-like thyroid neoplasms. **a** cGANs can create synthetic layer-blended images by conditioning the network using different embeddings at each layer. **b** Layer blending with a seed from the Thyroid cGAN reveals different morphologic changes associated with the *RAS*-like and *BRAF*^V600E^-like gene expression spectrum. Each image includes a corresponding classifier prediction, from -1 (*BRAF*^V600E^-like) to +1 (*RAS*-like). Image B1 is a fully *RAS*-like image, and Image B6 is a fully *BRAF*^V600E^-like image. Images B2-B5 are generated by using different class embeddings at each cGAN layer. Examining the resulting morphologic changes that occur when passing the *BRAF*^V600E^-like embedding to different layers illustrates different types of morphologic changes associated with the *BRAF*^V600E^-RAS spectrum.

Layer blending can provide deeper insights into class-specific morphology, as passing different embeddings to each layer in the cGAN offers a method for controlling the scale at which an image is influenced to be more like one class or another (Fig. 5a). We assessed the utility of layer blending to illustrate morphologic differences at difference scales using the *BRAF-RAS* gene expression endpoint. In Fig. 5b, a synthetic *RAS*-like image is shown as image B1, and a *BRAF*^V600E^-like image from the same seed is shown as image B6. Passing the *BRAF*^V600E^-like embedding only to layers 4–6, as shown in image B2, results in a decrease in size and variation in morphology of the follicles compared to image B1, but the prediction does not move in a *BRAF*^V600E^-like direction. Passing a *BRAF*^V600E^-like embedding to layers 7–9, as shown in Image B3, instead results in a minimal increase in chromatin clearing and a more eosinophilic color profile, and the classifier prediction has now moved closer to the *BRAF*^V600E^-like end of the spectrum. In image B4, setting layers 10-12 to *BRAF*^V600E^-like results in subtle changes to the stroma, resulting in a more ropey appearance to the collagen as well as a more eosinophilic color profile.

### Comparison with gradient-based pixel attribution

**Fig. 6:**
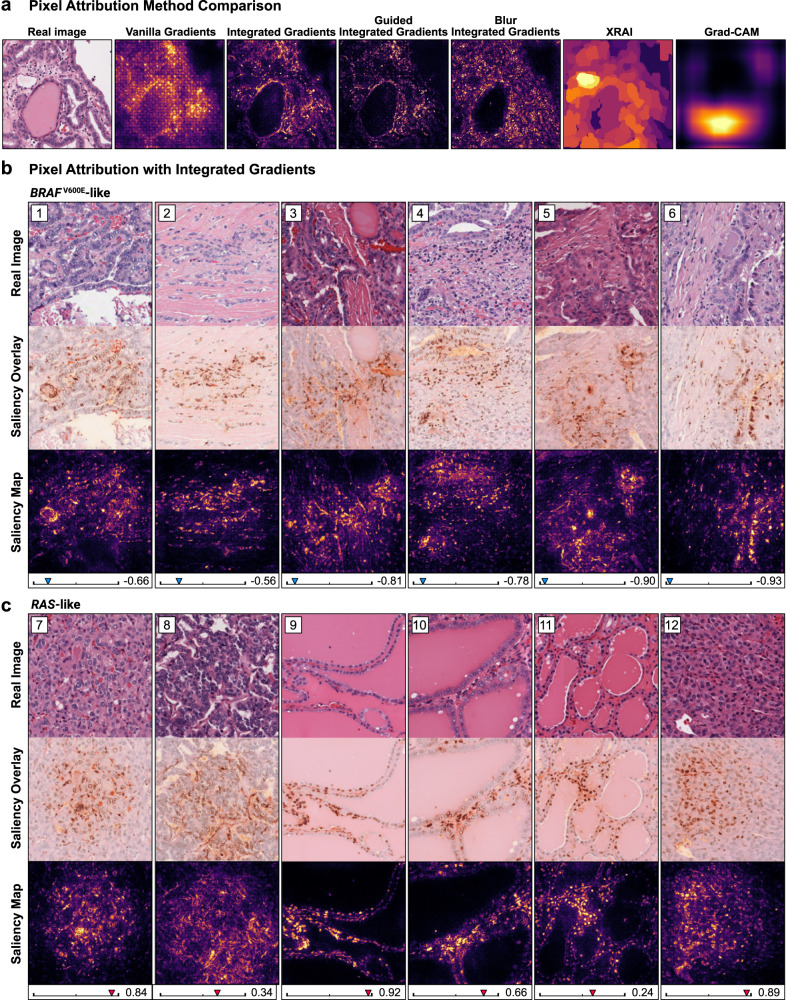
Local explainability with gradient-based pixel attribution. Twelve randomly sampled and correctly predicted real histologic images were taken from the thyroid *BRAF-RAS* training dataset, including six *BRAF*^V600E^-like images and six *RAS*-like images. Saliency maps were generated using pixel attribution calculated via integrated gradients. For each image, the associated classifier prediction is shown underneath. Saliency maps were reviewed with two domain-expert pathologists. **a** Comparison of different pixel attribution methods for a single real image. Grad-CAM, vanilla gradients, and XRAI methods yield coarse attribution maps that in general offer fewer insights than the more detailed attribution maps produced by methods based on integrated gradients. **b** Saliency maps for *BRAF*^V600E^-like images. The saliency map in image (1) emphasizes the nuclear membrane, sparing the cytoplasm. The importance of the papillary architecture seen in this image – often associated with *BRAF*^V600E^-like images, is not well conveyed. Image (2) highlights tumor cell nuclei and largely ignores stroma. Nuclei are again highlighted in (3), and notably the colloid does not have high attribution. In image (4), inflammatory cells are prominently highlighted. Image (5) shows high attribution in several densely eosinophilic, apoptotic bodies, which are associated with *BRAF*^V600E^-like status (likely reflecting high cell turnover). Image (6) shows greater attribution for dark, wrinkled, and irregular nuclei, and does not display high attribution for the surrounding stroma. **c** Saliency maps for *RAS*-like images. Image (7) highlights nuclear membranes and appears to outline vessels. Vessels are similarly outlined with high attribution in image (8) and (11). Images (9) and (11) show high attribution in nuclei. Image (10) highlights both the nuclear membrane and inflammatory cells. Images (11) and (12) also show high attribution in nuclei and interfollicular outlines. For all shown saliency maps, SmoothGrad^59^ was used to help reduce noise.

We used several gradient-based pixel attribution methods on the trained *BRAF-RAS* gene expression classifier to better understand the differences between explanations from these localization-based approaches and synthetic histology (Fig. 6). Unlike the synthetic histology approach, which provides dataset-level insights into morphologic features between classes, these gradient-based tools provide explanations through saliency maps that highlight areas of an image likely to be important for the classifier prediction. Grad-CAM, XRAI, and vanilla gradients yielded coarse saliency maps which offered little information for this histopathologic application (Fig. 6a). Integrated gradients, guided integrated gradients, and blur integrated gradients all generally yielded similar results. We generated saliency maps using integrated gradients for randomly sampled *BRAF*^V600E^-like and *RAS*-like images from the thyroid training dataset, and reviewed these maps with two domain-expert pathologists (Fig. 6b, c). These saliency maps identified relevant localizable image features known to be associated with the *BRAF-RAS* spectrum, including apoptotic bodies, denser eosinophilic cytoplasm, occasionally darker nuclei, or wrinkled and irregular nuclei in *BRAF*^V600E^-like images, and interfollicular outlines in the *RAS*-like images. However, the importance of larger and less localizable features, such as papillary architecture, stromal characteristics (such as vascularity versus desmoplasia), and colloid characteristics, were not well conveyed. Additionally, similar features were sometimes highlighted in both *BRAF*^V600E^-like and *RAS*-like images, such as nuclear membranes and inflammatory cells, making it difficult to interpret which class these features would be associated with.

### Educational intervention using synthetic histology

**Fig. 7:**
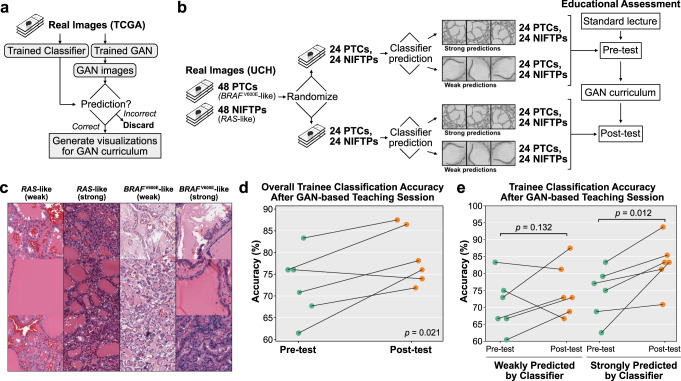
Synthetic histology augments pathologist-in-training education. **a** Schematic for creating GAN-based educational curriculum. A trained classifier and cGAN were used to generate and select classifier-concordant synthetic histology images, along with class blending videos generated through embedding interpolation. Synthetic histology images and videos were curated by an expert pathologist and incorporated into an educational curriculum. **b** Schematic for assessing effect of cGAN-based educational session on ability for pathology trainees to accurate classify images from real thyroid neoplasms. 48 *BRAF*^V600E^-like PTCs and 48 RAS-like NIFTPs were randomly split into a pre-test and post-test dataset. Predictions were generated for tiles from each slide using the trained BRS classifier and separated into weakly correct predictions (correct predictions between −0.5 and 0.5) and strongly correct predictions (correct predictions <−0.5 or > 0.5). For each slide, three weakly-correct images were randomly selected and merged into a single image trio, and three strongly-correct images were randomly selected and merged, resulting in 2 images per slide. The pre-test thus consisted of 96 images from 48 slides. The same procedure was taken for the post-test, comprised of 96 images from 48 different slides. **c** Example real image trios used during pre-test or post-test. **d** Trainee classification accuracy on real images significantly improved after the teaching session, from 72.7% to 79.0% (*p* = 0.021). **e** Improvement in trainee classification accuracy was greater for real images with strong classifier predictions (74.3% to 83.0%, *p* = 0.012) compared to real images with weak predictions (70.8% to 75.0%, *p* = 0.132).

Finally, we tested the use of synthetic histology to augment pathologist-in-training education by creating a cGAN-based educational curriculum illustrating the *BRAF-RAS* spectrum in thyroid neoplasms (Fig. 7). Six pathology residents first received a standard educational lecture on thyroid neoplasms, including discussion of NIFTP subtype and differences in *BRAF*^V600E^-like and *RAS*-like morphology. Residents completed a 96-question pre-test comprised of images of real tumors from a University of Chicago dataset, predicting whether images were *BRAF*^V600E^-like (PTC) or *RAS*-like (NIFTP). Residents then participated in a one-hour cGAN-based educational session, which included image pairs of synthetic *BRAF*^V600E^-like and *RAS*-like images generated from the same seed, video interpolations showing the gradual transition from *BRAF*^V600E^-like to *RAS*-like (Supplementary Data), and a computer-based interface in which residents could interactively generate synthetic images. Following the teaching session, residents completed a 96-question post-test comprised of real images from different cases than the pre-test. After the one-hour educational session, resident accuracy on real pathologic images significantly improved from 72.7% to 79.0% (*p* = 0.021, 95% CI for difference in means 1.7%–∞) (Fig. 7d).

## Discussion

The ability to understand how deep learning histopathological classifiers make their predictions will have broad implications for the interpretability and reliability of potential DNN biomarkers and may provide an avenue for discovering how biological states manifest morphologically.

Our results show that cGANs can be leveraged as an explainability method for histopathological DNN models, providing interpretable explanations for image features associated with classifier predictions through the generation of synthetic histology. For weakly supervised histopathological applications where tile-level image labels are inherited from the parent slide, not all labeled images in the training dataset are expected to possess image features associated with the outcome of interest, such as image tiles with background normal tissue, pen marks, out-of-focus areas, or artifact. GANs are trained to generate images from the entire training distribution, which includes these potentially uninformative image tiles. By filtering cGAN-generated images with classifier concordance, we use the classifier’s predictions to identify synthetic image pairs that are enriched for morphologic differences related to the outcome of interest. We demonstrate that this approach highlights known morphologic differences between lung adenocarcinoma and squamous cell carcinomas, ER-negative and ER-positive breast cancers, HPV-positive and HPV-negative head and neck squamous cell carcinomas, and *BRAF*^V600E^-like and *RAS*-like thyroid neoplasms. Generating class-blended images which smoothly transition from one class to another through linear interpolation of the embedding latent space further improves intuitiveness of the synthetic image explanations, and assists with interpreting differences between image pairs when the images are markedly different from one another.

This dataset-label explainability approach has some key advantages over other local explainability methods for histopathological models. Gradient-based pixel attribution methods, such as Grad-CAM and integrated gradients, can highlight morphologically significant image features relevant to classifier predictions for a specific image. These methods are, however, unable to convey differences between image classes that do not easily localize within an image, such as broad architectural changes, variations in hue and color, staining differences, and changes in stromal characteristics (Fig. 6). In contrast, synthetic histology generated by cGANs offer complementary insights into potentially subtle morphologic differences between image classes. Compared with TCAV and image captioning, this approach does not require a priori specification of captions or concepts thought to be important for prediction, capturing a broader array of morphologic differences and permitting more exploratory use. Finally, the approach does not require any additional labeled training data, instead utilizing the same training distribution used for the classifier being explained. When combined with other explainability approaches, class- and layer-blending with cGANs can improve richness of classifier explanations and support biological plausibility of DNN predictions.

cGAN-generated synthetic histology also provides an avenue for hypothesis generation through illustration of morphologic patterns capable of being learned by deep learning. The application of this method in thyroid neoplasms is a particularly salient example: a DNN trained to predict a gene expression signature and a cGAN conditioned on the same signature together illustrate the morphologic manifestation of *BRAF*^V600E^-like and *RAS*-like gene expression. This not only provides a method of explaining the DNN classifier – it also provides insights into how underlying tumor biology is connected with morphologic phenotype. A similar approach could be used to investigate morphologic manifestations of other molecular states in any cancer.

In addition to its use for DNN explainability, this approach provides a potential tool for pathology trainee education, particularly for rare tumors or elusive diagnoses. cGANs can generate synthetic histology that, with curation by an expert pathologist, depict subtle differences significant for diagnosis that may otherwise be challenging to clearly demonstrate with real histopathological images. The generation process can be both controlled and fine-tuned, allowing an educator to build a curriculum using synthetic histologic images for a particular objective or phenomenon as a supplement to real images. In our small study with pathology residents at a single institution, a short education curriculum utilizing synthetic histology improved trainee recognition of diagnostically challenging thyroid neoplasms. It is important to emphasize that this small educational intervention was intended as a proof-of-concept to explore the potential utility of the approach; the study was not powered to disentangle the benefit of synthetic histology on learning from improvements due to repeat content exposure.

Although synthetic histology offers compelling possibilities for both DNN explainability and trainee education, some important limitations must be acknowledged. As an explainability tool, cGANs provide global explanations using synthetic image examples. Thus, their utility is in improving understanding at the dataset level, rather than providing local insights into why a prediction was made for a specific real image. It also requires training a separate GAN model, incurring additional computational time and requiring a modest training dataset size which may be challenging to obtain for some clinical outcomes or very rare tumors. A well-performing classification model is also necessary for this cGAN-based explainability pipeline, as differences in classifier architectures and training paradigms may impact the selection of GAN seeds used for generating synthetic histology explanations. Finally, GANs may learn to generate images that reflect underlying biases in the training dataset. Identification of bias is advantageous for explainability, as it may assist with highlighting potential confounding factors such as stain or color differences. Careful curation by an expert pathologist will be required to utilize synthetic histology for education in order to prevent perpetuation of potential biases in the training dataset.

In summary, cGANs can generate realistic, class-specific histologic images, and exploring visualizations from images with high classifier concordance provides an intuitive tool for deep learning model explainability. Class blending via embedding interpolation yields realistic images with smooth transitions between classes, and layer blending reveals unique morphological constructs at architectural, cellular, and stromal levels. Synthetic histology not only offers an approach to model explainability, but can also provide new, hypothesis-generating insights into histologic associations with molecularly-defined tumor subtypes. Finally, synthetic histology can also be an effective teaching aid, capable of improving trainee recognition of histologic classes in a rare cancer subtype.

## Methods

### Dataset description

The Lung cGAN was trained on 941 whole-slide images (WSI) from The Cancer Genome Atlas (TCGA), including 467 slides from the lung adenocarcinoma project (TCGA-LUAD) and 474 slides from the lung squamous cell carcinoma project (TCGA-LUSC) (https://portal.gdc.cancer.gov/). Validation was performed on 1306 WSIs from the Clinical Proteomic Tumor Analysis Consortium (CPTAC) lung adenocarcinoma (CPTAC-LUAD) and lung squamous cell carcinoma (CPTAC-LSCC) collections (https://www.cancerimagingarchive.net/collections/). The Breast cGAN was trained on 1,048 WSIs from The Cancer Genome Atlas (TCGA-BRCA), including 228 estrogen receptor (ER) negative tumors and 820 ER-positive tumors. Validation was performed on 98 WSIs from CPTAC, including 26 ER-negative and 72 ER-positive tumors, with ER status determined through IHC staining using standard clinical criteria. The Head and Neck cGAN and classifiers were trained on 362 WSIs from a single-site institutional dataset, including 202 HPV-negative and 160 HPV-positive tumors. HPV status on the institutional dataset was determined through positivity by either PCR or p16 IHC. Validation was performed on 405 WSIs from TCGA (TCGA-HNSC), with 359 HPV-negative and 46 HPV-positive tumors. The Thyroid cGAN was trained on 369 WSIs from The Cancer Genome Atlas (TCGA-THCA), including 116 *BRAF*^V600E^-like tumors (where *BRAF-RAS* gene expression score is less than 0) and 271 *RAS*-like tumors (where *BRAF-RAS* gene expression score is greater than 0). Validation was performed on an institutional dataset of 134 tumors, including 76 *BRAF*^V600E^-like PTCs and 58 *RAS*-like NIFTPs.

### Image processing

For the classifier models, image tiles were extracted from WSIs with an image tile width of 302 μm and 299 pixels using Slideflow version 1.3.1^50^. For the breast and lung cGANs, image tiles were extracted with an image tile width of 400 μm and 512 pixels. For the thyroid cGAN, image tiles were extracted at 302 μm and 512 pixels. Background was removed via grayspace filtering, Otsu’s thresholding, and gaussian blur filtering. Gaussian blur filtering was performed with a sigma of 3 and threshold of 0.02. Image tiles were extracted from within pathologist-annotated regions of interest (ROIs) highlighting areas of tumor, in order to remove normal background tissue and maximize cancer-specific training.

### Classifier training

We trained deep learning classification models based on an Xception architecture, using ImageNet pretrained weights and two hidden layers of width 1024, with dropout (*p* = 0.1) after each hidden layer. Xception was chosen out of prior experience due to its fast convergence and high performance for histopathological applications^43,51–54^. Models were trained with Slideflow using the Tensorflow backend with a single set of hyperparameters and category-level mini-batch balancing (Supplementary Table 3). Training images were augmented with random flipping and cardinal rotation, JPEG compression (50% chance of compression with quality level between 50 and 100%), and gaussian blur (10% chance of blur with sigma between 0.5 and 2.0). Random Gaussian blurring is a technique used to simulate of out-of-focus artifacts, a common issue encountered when scanning slides with whole-slide image scanners, and may theoretically improve performance and generalizability^55,56^. Training images also underwent stain normalization with a modified Reinhard^57^ method, with the brightness standardization step removed for computational efficiency. Binary categorization models (lung and breast classifiers) were trained with cross-entropy loss, and the thyroid BRS classifier was trained with mean squared error loss. Models were first trained with site-preserved cross-validation^52^, then a final model was trained across the full dataset and validated on an external dataset. Classifier models were evaluated by calculating Area Under Receiver Operator Curve (AUROC), with cross-validation AUROC reported as mean ± SD. AUROC 95% confidence intervals and p-values were calculated with the DeLong method^58^.

### cGAN training

Our cGAN architecture is an implementation of StyleGAN2, minimally modified to interface with the histopathology deep learning package Slideflow and allow for easier embedding space interpolation^14^. The lung cGAN was conditioned on the binary category of adenocarcinoma vs. squamous cell carcinoma, and the breast cGAN was conditioned on the binary category of ER-negative vs. ER-positive. The thyroid cGAN was conditioned on a binary categorization of the continuous *BRAF-RAS* score, discretized at 0 into *BRAF*^V600E^-like (less than 0) or *RAS*-like (greater than 0). All cGANs were trained on images without stain normalization, to improve training dataset diversity and aid in pathologist interpretation of raw images without normalization artifacts.

The lung cGAN was trained on 4 A100 GPUs for 25,000 kimg (25 million total images) starting with random weight initialization. The breast cGAN was trained on 4 A100 GPUs for 10,000 kimg (10 million total images), and the thyroid cGAN was trained on 2 A100 GPUs for 12,720 kimg (12.7 million total images), stopped at this time point due to model divergence with further training. All cGANs were trained with an R1 gamma of 1.6384, batch size of 32, and using all available augmentations from StyleGAN2. cGANs were evaluated by calculating Fréchet Inception Distance (FID)^37^ using the full real image dataset and 50,000 cGAN-generated images.

### Classifier concordance

To assess classifier concordance for a cGAN and an associated classifier, the cGAN generates an image for each class using the same seed. The generated images are center-cropped, resized to match the same histologic magnification as the associated classifier, and stain normalized using the modified Reinhard method. and the classifier creates predictions for each image. Predictions are considered “strong” if the post-softmax value is greater than 0.75 for the predicted class, and “weak” if the post-softmax value for the predicted class is less than 0.75. For the thyroid BRS classifier which uses a continuous outcome, predictions are considered “strong” if the raw prediction is less than −0.5 or greater than 0.5, and “weak” if the prediction is between -0.5 and 0.5. A given seed is defined as strongly concordant if the classifier predictions match the cGAN class labels for both images and the predictions are both strong. A seed is weakly concordant if the classifier predictions match the cGAN class labels, but either prediction is weak. A seed is non-concordant if the classifier predictions do not match the cGAN class labels.

### cGAN class and layer blending

To create class-conditional images, cGAN class labels are projected into an embedding space before conditioning the network, with the projection learned during training. After training, each class label has a single associated embedding vector. To create class-blended images, we perform a linear interpolation between class embeddings and use these interpolated embeddings for network conditioning while holding the cGAN seed constant. We create layer-blended images by passing different class embeddings to each cGAN network layer while holding the cGAN seed constant.

### Pathologist assessment of cGAN images

Domain-expert pathologists reviewed at least 50 strongly-concordant synthetic histologic images to assess realism, variety, and consistency with cGAN class labels. Pathologists first reviewed the images in a blinded fashion without knowledge of the associated cGAN labels. Lossless, PNG images were viewed at the full 512 ×512 px resolution. Pathologists then reviewed the strongly-concordance synthetic image pairs side-by-side with knowledge of the cGAN labels to assess consistency of the synthetic images with biological expectations for the associated class labels. Pathologists described histologic differences between each image pairs and provided an overall summary of thematic differences between classes.

### cGAN educational session

Six pathology were recruited for this study via email. No sample-size calculation was performed prior to recruitment. Participating residents received a one-hour lecture as a part of their core educational curriculum discussing the histopathological diagnosis of thyroid neoplasms, including a discussion of differentiating between malignant papillary thyroid carcinomas (PTCs), including follicular-variant PTCs, and benign non-invasive follicular thyroid neoplasms with papillary-like nuclear features (NIFTP). A discussion of the molecular association between PTCs and *BRAF*^V600E^ mutations, and NIFTPs and *RAS* mutations, was also included.

Pathology residents then took a pre-test based on 96 real images from 48 cases at the University of Chicago, including 24 PTCs (both classic and follicular-variant) and 24 NIFTPs. The trained BRS classifier model generated predictions across all whole-slide images, and for each case, three strongly-predicted image tiles (prediction less than −0.5 or greater than 0.5) were randomly selected and merged side-by-side, and three weakly-predicted image tiles (prediction between −0.5 and 0.5) were randomly selected and merged, resulting in two merged image trios for each of the 48 cases. The pre-test was comprised of weak and strong image trios for 24 PTCs and NIFTPs, and residents were asked to predict whether the image trios came from a *BRAF*^V600E^-like tumor (PTC) or *RAS*-like tumor (NIFTP).

Residents then participated in a one-hour cGAN-based educational curriculum. The curriculum was developed by first calculating classifier concordance for 1000 seeds and identifying the strongly-concordant seeds. *BRAF*^V600E^-like and *RAS*-like image pairs for the 412 strongly-concordant seeds were reviewed by a domain expert pathologist. The vast majority of these images highlighted morphologic features known to be associated with the *BRAF-RAS* spectrum, and a diverse subset of 46 were chosen for inclusion in the teaching session. Video interpolations were generated and shown for seven of these seeds. The educational session was structured as a PowerPoint presentation, using only synthetic histologic image pairs and video interpolations to highlight important morphologic differences associated with the BRS spectrum. Residents also had access to a computer workstation loaded with an interactive visualization of cGAN generated images and class blending, to supplement the visualizations shown in slideshow format. Synthetic images shown on the workstation were displayed both pre- and post- stain normalization.

Finally, residents completed a post-test comprised of 96 images from 48 different cases than the pre-test, and resident classification accuracy was compared using a one-sided paired T-test.

### Ethics statement

Educational study was reviewed by the Biological Sciences Division/University of Chicago Medical Center Institutional Review Board and deemed minimal risk, exempt from protocol approval and requirement for informed consent.

### Reporting summary

Further information on research design is available in the Nature Research Reporting Summary linked to this article.

## Supplementary information

Supplementary Information

Supplementary Data: Accession numbers and annotations for TCGA data.

REPORTING SUMMARY

## Data Availability

All data and associated accession numbers used for training is included in this repository, and can be additionally accessed directly at https://portal.gdc.cancer.gov/ and https://www.cancerimagingarchive.net/collections/ using the accession numbers provided in the **Supplementary Data**. Restrictions apply to the availability of the internal University of Chicago thyroid dataset, but all requests will be promptly evaluated based on institutional and departmental policies to determine whether the data requested are subject to intellectual property or patient privacy obligations. The University of Chicago dataset can only be shared for non-commercial academic purposes and will require a data user agreement. Class interpolation videos generated using the thyroid cGAN are available at 10.5281/zenodo.7921816.

## Code availability

All code and models are made publicly available with an interactive user interface for class blending and latent space navigation at https://github.com/jamesdolezal/synthetic-histology, which includes all code necessary for reproducing results of this manuscript. The user interface provided is the same interface used during the educational teaching session. This code utilizes the software package Slideflow, version 1.3.1^50^, available at https://github.com/jamesdolezal/slideflow.
