# Supplementary material for: Deep learning generates synthetic cancer histology for explainability and education
Source: NPJ Precis Oncol. 2023 May 29;7:49. doi: 10.1038/s41698-023-00399-4 (PMC10227067; doi:10.1038/s41698-023-00399-4)
Supplement: Supplementary file 1 — Supplementary Information [file 41698_2023_399_MOESM1_ESM.pdf]

Table of Contents

Supplementary Figure 1 .... **2**
Supplementary Figure 2 .... **3**
Supplementary Figure 3 .... **5**
Supplementary Figure 4 .... **7**
Supplementary Figure 5 .... **9**
Supplementary Figure 6 .... **10**
Supplementary Figure 7 .... **11**
Supplementary Table 1 .... **12**
Supplementary Table 2 .... **13**
Supplementary Table 3 .... **14**

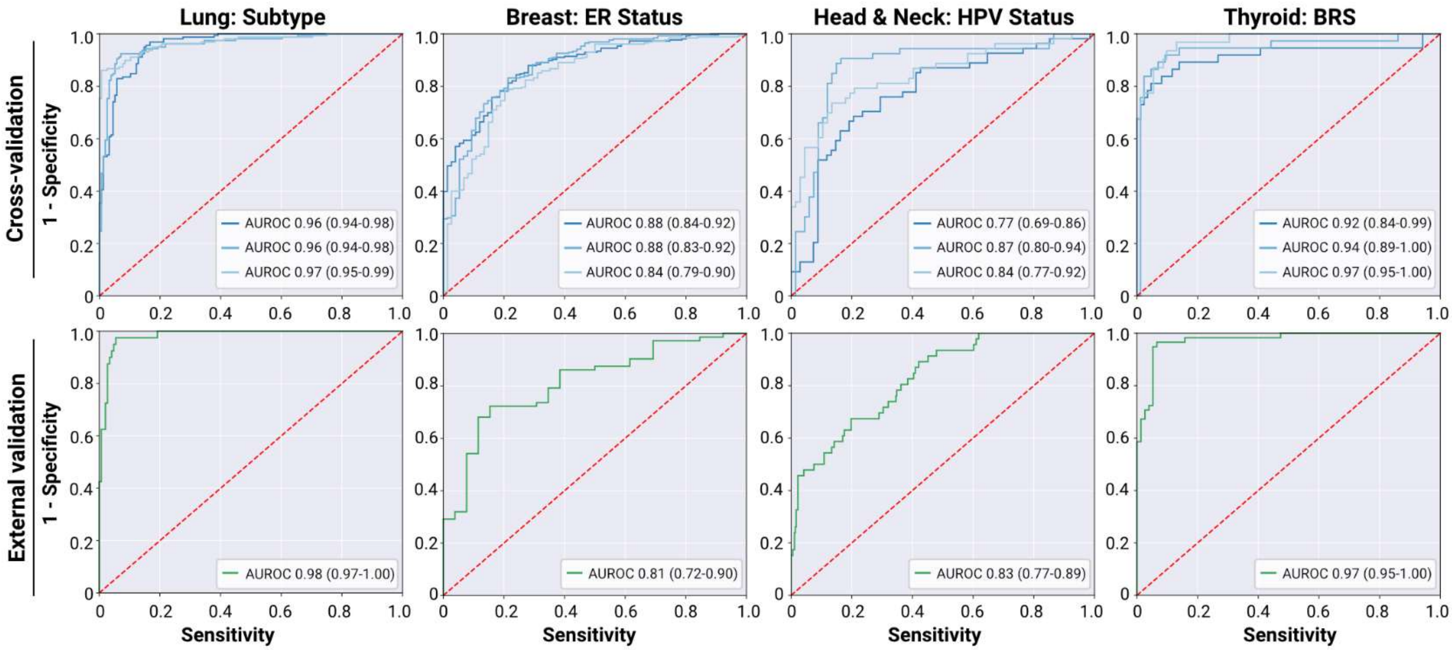

Supplementary Figure 1. Cross-validation and external validation results for the classification models. Classification models were trained for lung cancer predicting adenocarcinoma vs. squamous cell carcinoma, breast cancer predict estrogen receptor (ER) status, and thyroid neoplasms predicting BRAF-RAS Score (BRS). All models were first trained on data from The Cancer Genome Atlas (TCGA) using site-preserved cross-validation to reduce institutional bias. Following cross-validation, a final model for each tumor type was trained across the full dataset, and validated on the external, held-out validation dataset. The external datasets for the lung and breast models were comprised of 1,306 and 98 slides from the Clinical Proteomic Tumor Analysis Consortium (CPTAC), respectively. The external dataset for the thyroid model was comprised of 134 tumors from University of Chicago. For the thyroid BRS outcome, models were trained to predict BRS as a continuous outcome; the AUROCs shown here are a result of discretizing the BRS predictions into BRAF-like (less than 0) and RAS-like (greater than 0). AUROCs are shown with 95% confidence intervals obtained via the DeLong method. P-values were calculated with the DeLong method comparing against an AUROC of 0.5, and P-values for all AUROCs shown were <0.0001. AUROC = Area Under Receiver Operator Curve.

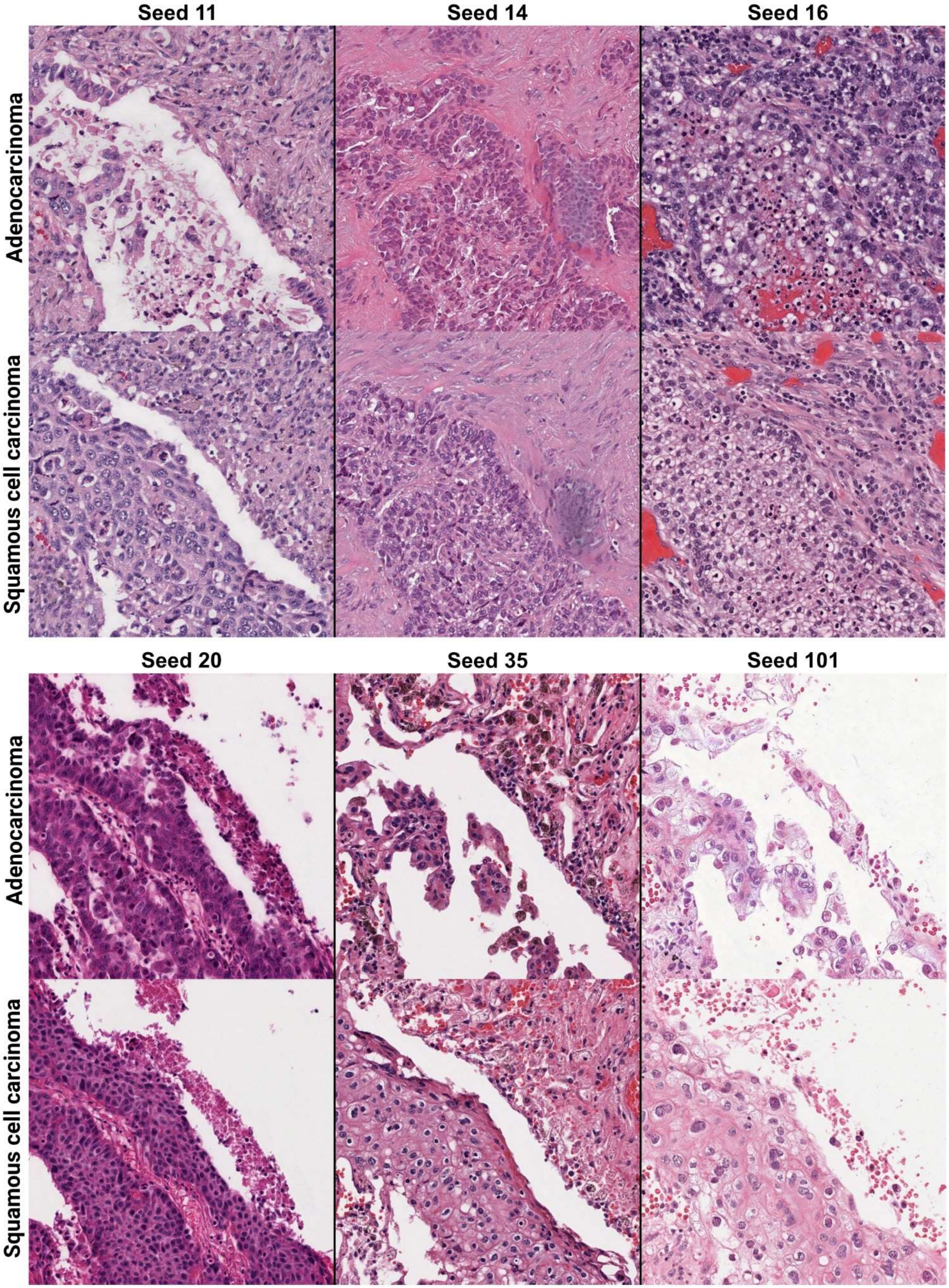
Seed 11
Seed 14
Seed 16
Adenocarcinoma
Squamous cell carcinoma
Seed 20
Seed 35
Seed 101
Adenocarcinoma
Squamous cell carcinoma

Supplementary Figure 2. cGAN-generated images from a model conditioned on lung cancer adenocarcinoma vs. squamous cell carcinoma. Classifier-concordant seeds were reviewed with an expert thoracic pathologist and pathology fellow to assess differences in cGAN-generated adenocarcinoma and squamous cell images, and example image pairs for select seeds are shown here. Images are shown without stain normalization. For seed 11, the adenocarcinoma image shows clear glandular formation, and the squamous cell image has hints of intercellular bridges consistent with squamous morphology, although the bridging is subtle at this power. For seed 14, the adenocarcinoma image shows hints at gland formation, whereas the squamous cell image is more solid. Several foci of intercellular bridges are seen in the squamous cell image. In seed 16, the adenocarcinoma image is suggestive of papillary architecture which could be consistent with adenocarcinoma, and the squamous cell image has greater clear cells which are associated with squamous morphology. The adenocarcinoma image for seed 20 shows glandular formation, and the squamous cell image is clearly squamous with solid tumor and intercellular bridges. In seed 35, the adenocarcinoma image shows a papillary projection consistent with adenocarcinoma, and the squamous cell image shows hard cellular borders consistent with squamous morphology. Similarly, the adenocarcinoma image in seed 101 shows micropapillary morphology with a papillary projection, while the squamous cell image shows keratinization consistent with squamous morphology.

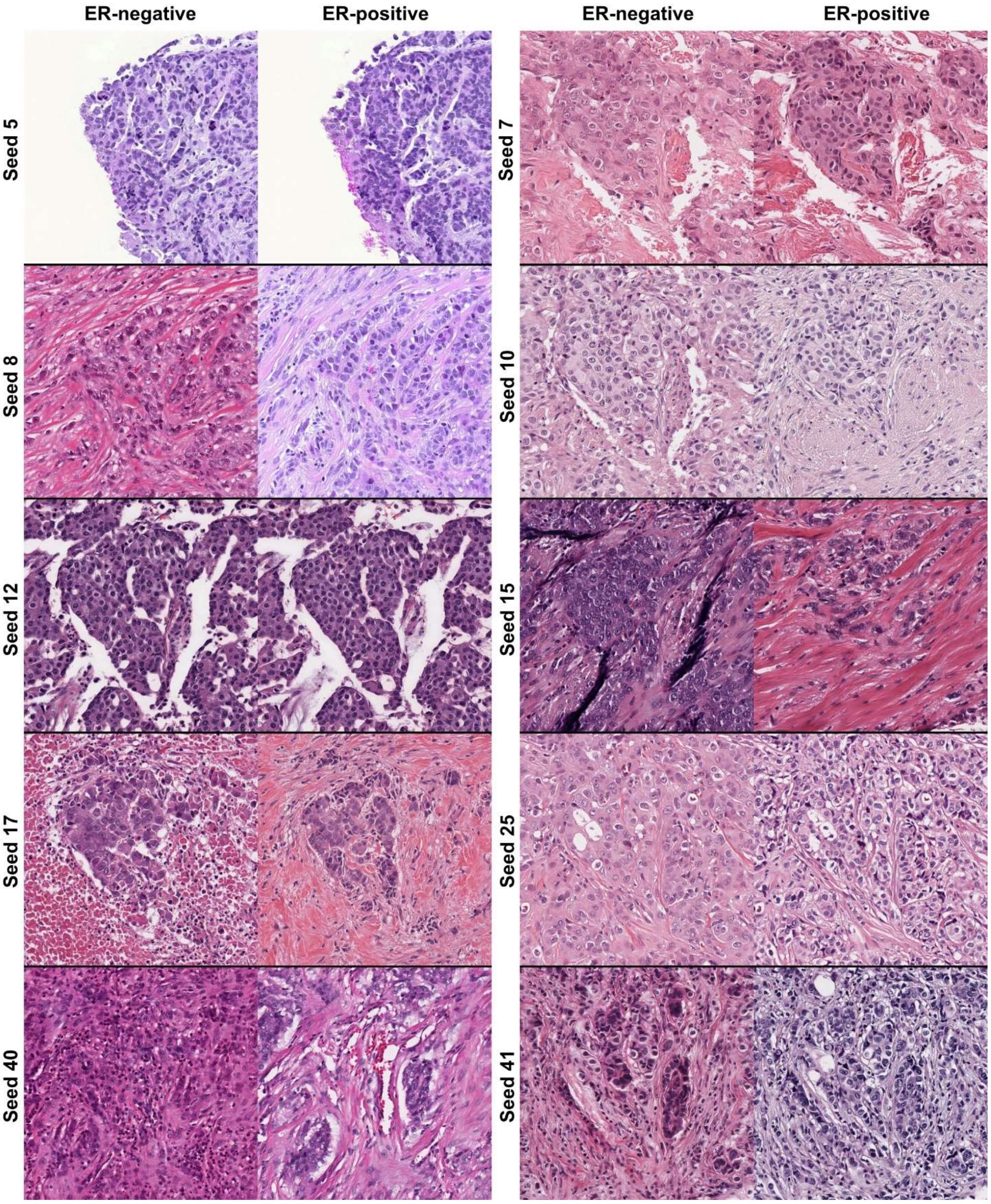
ER-negative
ER-positive
ER-negative
ER-positive
Seed 5
Seed 7
Seed 8
Seed 10
Seed 12
Seed 15
Seed 17
Seed 25
Seed 40
Seed 41

Supplementary Figure 3. cGAN-generated images from a model conditioned on breast cancer estrogen receptor (ER) status. Seeds with strong classifier concordance were reviewed with two expert breast pathologists to determine thematic differences in cGAN-generated ER-negative and ER-positive histologic features. Images are shown without stain normalization. The seed 5 ER-negative image has slightly higher grade than the ER-positive image, with greater mitotic activity and larger nuclei. Seed 7 similarly illustrates slightly higher grade in the ER-negative nuclei, with slight differentiation and duct formation in the ER-negative image. The ER-negative image from seed 8 shows slightly more open chromatin compared with ER-positive. Both images from seed 10 are higher grade, with the ER-positive image showing slight gland formation. The images from seed 12 are both very similar, but the ER-negative image shows necrotic debris and degenerative changes. In seed 15, the ER-negative image appears to be invasive ductal carcinoma (IDC), whereas the ER-positive image is more consistent with mixed lobular/ductal. Seed 17 shows nearly identical tumor cells in the ER-negative and ER-positive images with similar grade, but with more necrosis in the ER-negative tumor. Seed 25 illustrates slight apocrine differentiation in the ER-negative image compared with the ER-positive image. Seeds 40 and 41 both show increase in tumor-infiltrating lymphocytes in the ER-negative image compared with ER-positive, with slightly higher grade nuclei in the ER-negative image in seed 41.

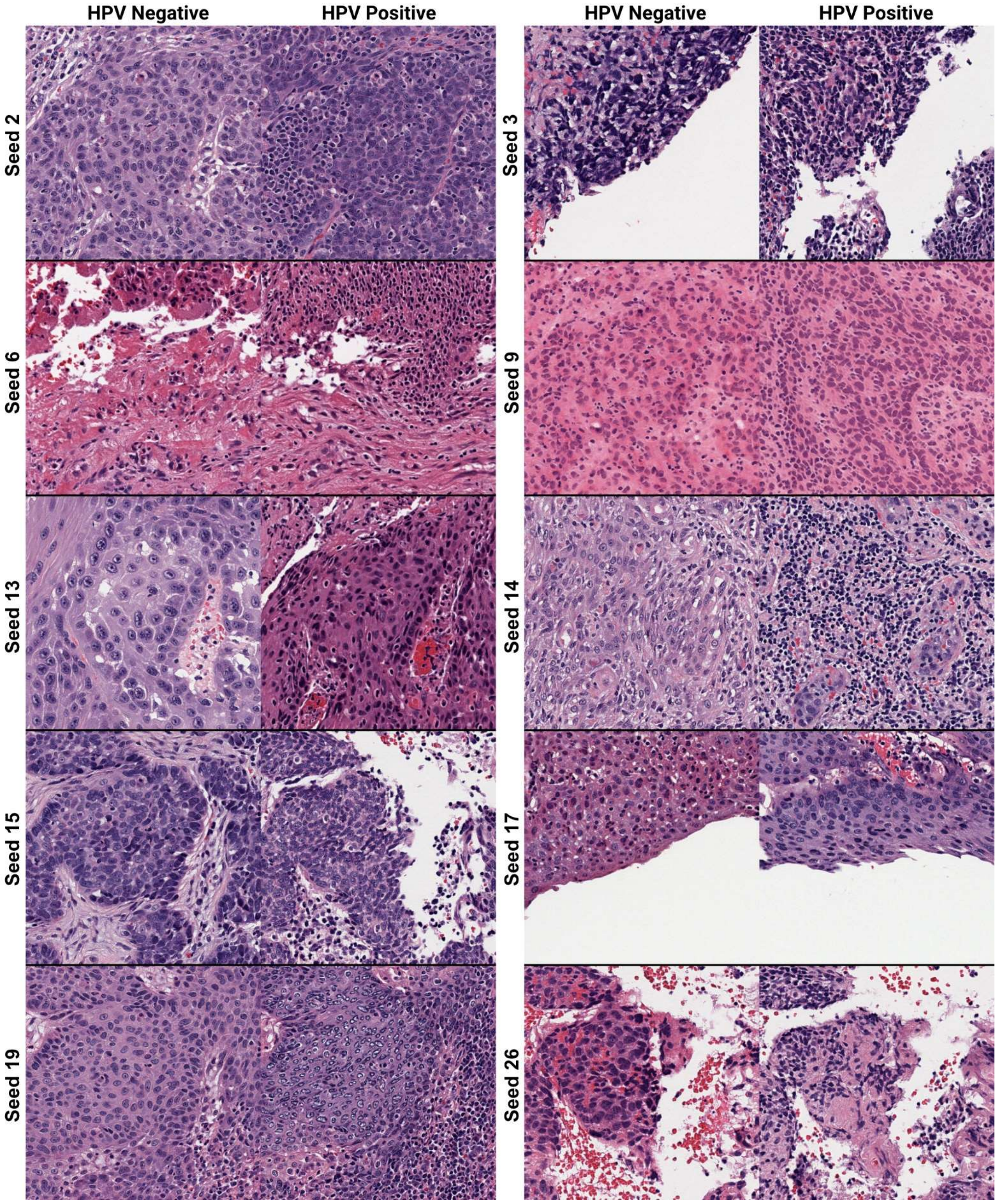

Supplementary Figure 4. cGAN-generated images from a model conditioned on head and neck cancer Human Papilloma Virus (HPV) status. Seeds with strong classifier concordance were reviewed with two domain expert pathologists to determine thematic differences in cGAN-generated HPV-negative and HPV-positive histologic features. Images are shown without stain normalization. In seeds 2 and 6, HPV-negative tumor cells have greater cytoplasm and

pleomorphic nuclei, whereas HPV-positive images show syncytial cells with scant cytoplasm in close proximity and surrounding inflammatory infiltrate. Seed 9 shows degenerating tumor cells in both images, but cells in the HPV-negative image have more eosinophilic tincture suggestive of dying keratin. In seed 13, color differences are seen due to apparent differences in staining. The HPV-negative image appears to show desmosomes between cells, which is related to keratinization status, and the HPV-positive tumor cells are more syncytial. Seed 14 is notable for significant inflammation in the HPV-positive image. Cells in the HPV-negative image have more foamy cytoplasm. For seed 15, the desmoplastic stroma seen in the HPV-negative image is replaced with fragmentation or dead space, and the HPV positive cells are smaller with decreased cytoplasm. The HPV-positive image in seed 17 displays more brightly eosinophilic cytoplasm compared to the HPV-negative image. Seed 19 illustrates inflammatory infiltrate and more syncytial cells in the HPV positive image. The HPV negative image in seed 26 shows large, more pleomorphic nuclei compared to the monotonous nuclei in the HPV positive image. In both seeds 15 and 19, the HPV-negative images show what appears to be peripheral palisading at the edge of tumor nests, which is not known to be a feature associated with HPV-negative status. All other described features, apart from staining differences, are consistent with known histopathologic associations with HPV status as seen on routine hematoxylin & eosin (H&E) stained slides. Some seeds, however, did not illustrate meaningful differences between classes. For example, the subtle differences in nuclear pleomorphism between seed 3 images are difficult to appreciate due to the appearance of crush artifact.

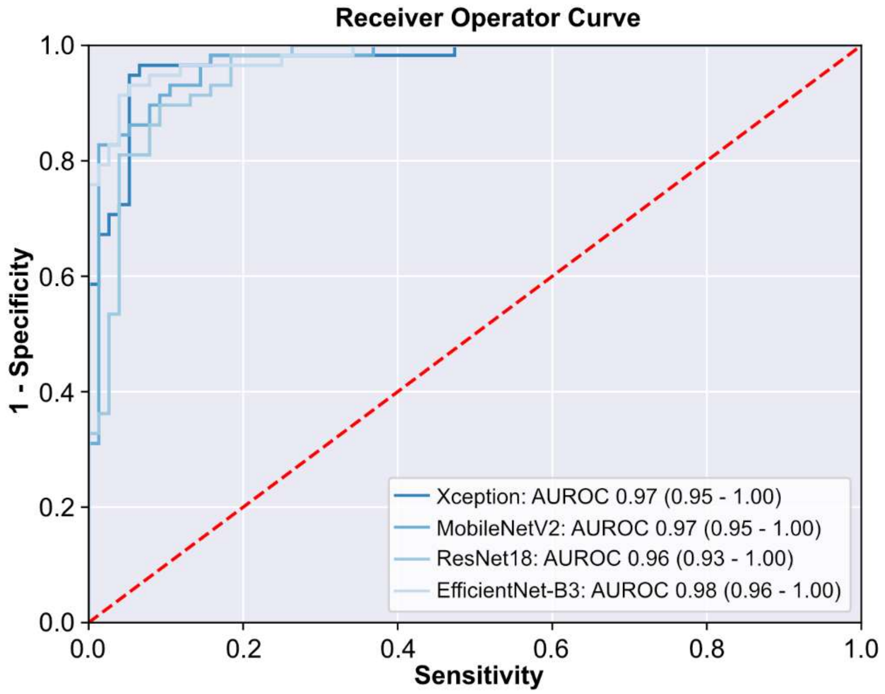

Supplementary Figure 5. Comparison of classifier architectures for prediction of thyroid BRAF-RAS gene expression score. To assess the potential impact of classifier architecture selection, we tested three alternative architectures against our standard prespecified Xception architecture, including MobileNetV2, ResNet18, and EfficientNet-B3. Classifiers were trained on the full TCGA dataset of thyroid neoplasms with BRAF-RAS score as a linear outcome. Classifiers were validated on an external institutional dataset of *BRAF*^{V600E}-like papillary thyroid carcinomas (PTCs) and *RAS*-like non-invasive follicular thyroid neoplasms with papillary-like nuclear features (NIFTP). Linear predictions were discretized at 0, with predictions less than 0 indicating *BRAF*^{V600E}-like and greater than 0 *RAS*-like. AUROC on the external test sets was similar between all architectures. The range of AUROCs displayed is a 95% confidence interval calculated with the DeLong method.

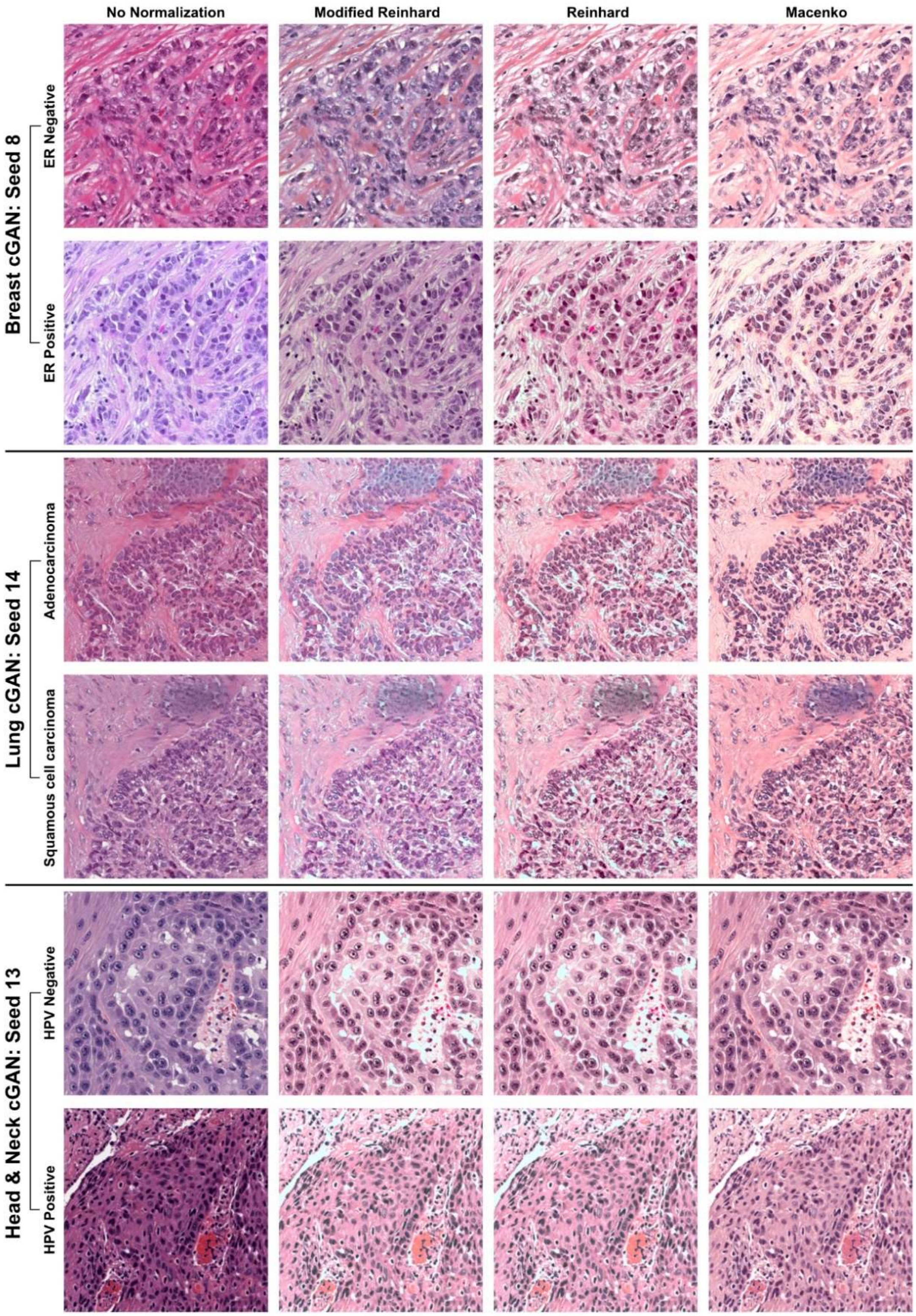

Supplementary Figure 6. Comparison of stain normalization methods on cGAN-generated synthetic images. Three example strongly-concordant synthetic images with visible staining differences are shown before and after stain normalization using three different methods. The first column shows images without stain normalization, as shown to pathologists during interpretation. The second column shows the image after normalization with the modified Reinhard method, the stain normalization algorithm used when preprocessing images for the classifiers. The third and fourth columns show the images after stain normalization using the standard Reinhard and Macenko methods, for comparison. In general, all stain normalization methods displayed similar efficacy in visually reducing differences in staining.

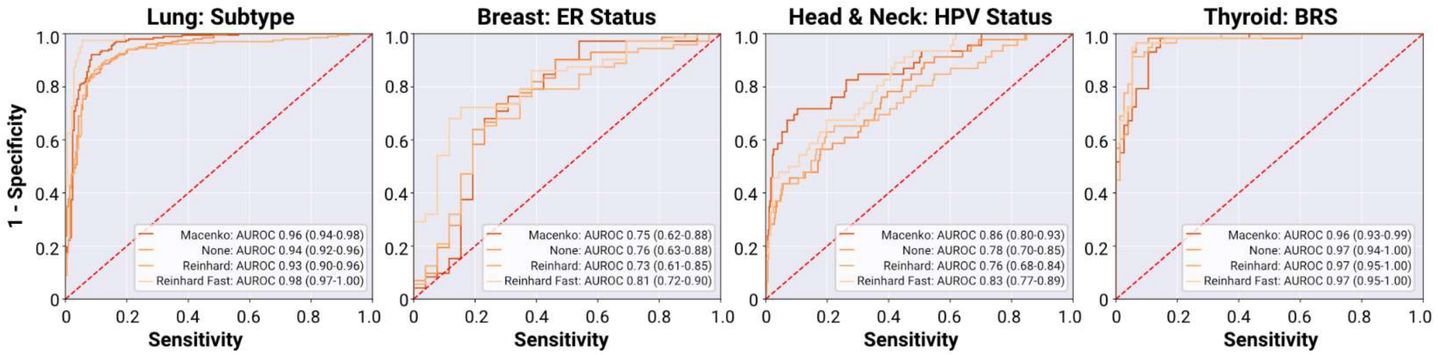

Supplementary Figure 7. Comparison of stain normalizations strategies for prediction of thyroid BRAF-RAS gene expression score. To assess the potential impact of stain normalization method on classifier performance, we tested three alternative stain normalization strategies – Reinhard, Macenko, and no normalization - against our standard prespecified Reinhard Fast method, a modification of the base Reinhard algorithm with the brightness standardization step removed. Classifiers were trained on the full TCGA dataset of thyroid neoplasms with BRAF-RAS score as a linear outcome. Classifiers were validated on an external institutional dataset of *BRAF*^{V600E}-like papillary thyroid carcinomas (PTCs) and *RAS*-like non-invasive follicular thyroid neoplasms with papillary-like nuclear features (NIFTP). Linear predictions were discretized at 0, with predictions less than 0 indicating *BRAF*^{V600E}-like and greater than 0 *RAS*-like. AUROC on the external test sets is shown above, with the range of AUROCs displayed as a 95% confidence interval calculated using the DeLong method.

Outcome	Model	AUROC	Average Precision	Balanced Accuracy
**Lung, subtyping**	Cross-fold 1	0.957 (0.937 - 0.977)	0.959	0.898
	Cross-fold 2	0.958 (0.936 - 0.980)	0.957	0.924
	Cross-fold 3	0.968 (0.950 - 0.986)	0.967	0.924
	External validation	0.983 (0.968 - 0.998)	0.968	0.948
**Breast, ER status**	Cross-fold 1	0.879 (0.839 - 0.919)	0.826	0.8
	Cross-fold 2	0.878 (0.834 - 0.923)	0.846	0.807
	Cross-fold 3	0.843 (0.791 - 0.895)	0.775	0.789
	External validation	0.812 (0.721 - 0.904)	0.768	0.777
**Head and neck, HPV status**	Cross-fold 1	0.775 (0.689 - 0.861)	0.752	0.73
	Cross-fold 2	0.874 (0.803 - 0.944)	0.843	0.861
	Cross-fold 3	0.845 (0.772 - 0.918)	0.846	0.791
	External validation	0.827 (0.768 - 0.887)	0.745	0.727
**Thyroid, *BRAF-RAS* Score**	Cross-fold 1	0.918 (0.845 - 0.991)	0.92	0.869
	Cross-fold 2	0.945 (0.890 - 1.000)	0.914	0.899
	Cross-fold 3	0.973 (0.948 - 0.998)	0.965	0.903
	External validation	0.973 (0.950 - 0.997)	0.974	0.941

Supplementary Table 1. Classifier performance metrics in cross-validation and external validation. Area Under Receiver Operator Curve (AUROC) values are shown with a 95% confidence interval obtained via the DeLong method.

Outcome	Model	AUROC	Average Precision	Balanced Accuracy
**Lung, subtyping**	Macenko normalization	0.960 (0.941 - 0.979)	0.956	0.916
	No normalization	0.944 (0.923 - 0.965)	0.944	0.88
	Reinhard normalization	0.930 (0.903 - 0.956)	0.921	0.883
	Modified Reinhard normalization	0.983 (0.968 - 0.998)	0.968	0.948
**Breast, ER status**	Macenko normalization	0.750 (0.620 - 0.880)	0.731	0.721
	No normalization	0.756 (0.634 - 0.878)	0.739	0.726
	Reinhard normalization	0.727 (0.608 - 0.846)	0.678	0.716
	Modified Reinhard normalization	0.812 (0.721 - 0.904)	0.768	0.777
**Head and neck, HPV status**	Macenko normalization	0.864 (0.802 - 0.926)	0.802	0.796
	No normalization	0.776 (0.705 - 0.846)	0.689	0.683
	Reinhard normalization	0.758 (0.676 - 0.839)	0.714	0.705
	Modified Reinhard normalization	0.827 (0.768 - 0.887)	0.745	0.727
**Thyroid, *BRAF-RAS* Score**	Macenko normalization	0.960 (0.931 - 0.988)	0.96	0.91
	No normalization	0.970 (0.942 - 0.998)	0.969	0.941
	Reinhard normalization	0.973 (0.950 - 0.996)	0.972	0.922
	Modified Reinhard normalization	0.973 (0.950 - 0.997)	0.974	0.941

Supplementary Table 2. Classifier performance metrics for models trained with varying stain normalization methods. Area Under Receiver Operator Curve (AUROC) values are shown with a 95% confidence interval obtained via the DeLong method.

Hyperparameter / Model Parameter	Value
augment	xyrjb
batch_size	128
dropout	0.1
early_stop	FALSE
epochs	1
hidden_layer_width	1024
hidden_layers	2
include_top	FALSE
l1	0
l1_dense	0
l2	0
l2_dense	0
learning_rate	0.0001
learning_rate_decay	0.98
learning_rate_decay_steps	512
loss	sparse_categorical_crossentropy
model	xception
normalizer	reinhard_fast
optimizer	Adam
pooling	avg
tile_px	299
tile_um	302
toplayer_epochs	0
trainable_layers	0
training_balance	category
uq	TRUE
validation_balance	None

Supplementary Table 3. Hyperparameters used during classifier training. The above hyperparameters were used for all classifiers during training, with the exception of the BRAF-RAS Score classifiers, which were trained with "mean_squared_error" loss.
